# CC‐chemokine receptor 7 (CCR7) deficiency alters adipose tissue leukocyte populations in mice

**DOI:** 10.14814/phy2.12971

**Published:** 2016-09-21

**Authors:** Jeb S. Orr, Arion J. Kennedy, Andrea A. Hill, Emily K. Anderson‐Baucum, Merla J. Hubler, Alyssa H. Hasty

**Affiliations:** ^1^ Department of Molecular Physiology and BiophysicsVanderbilt University School of MedicineNashvilleTennessee

**Keywords:** Adipose tissue, CCR7, macrophages, obesity, T cells

## Abstract

The mechanism by which macrophages and other immune cells accumulate in adipose tissue (AT) has been an area of intense investigation over the past decade. Several different chemokines and their cognate receptors have been studied for their role as chemoattractants in promoting recruitment of immune cells to AT. However, it is also possible that chemoattractants known to promote clearance of immune cells from tissues to regional lymph nodes might be a critical component to overall AT immune homeostasis. In this study, we evaluated whether CCR7 influences AT macrophage (ATM) or T‐cell (ATT) accumulation. CCR7^−/−^ and littermate wild‐type (WT) mice were placed on low‐fat diet (LFD) or high‐fat diet (HFD) for 16 weeks. CCR7 deficiency did not impact HFD‐induced weight gain, hepatic steatosis, or glucose intolerance. Although lean CCR7^−/−^ mice had an increased proportion of alternatively activated ATMs, there were no differences in ATM accumulation or polarization between HFD‐fed CCR7^−/−^ mice and their WT counterparts. However, CCR7 deficiency did lead to the preferential accumulation of CD8^+^ ATT cells, which was further exacerbated by HFD feeding. Finally, expression of inflammatory cytokines/chemokines, such as *Tnf*,* Il6*,* Il1β*,* Ccl2*, and *Ccl3*, was equally elevated in AT by HFD feeding in CCR7^−/−^ and WT mice, while *Ifng* and *Il18* were elevated by HFD feeding in CCR7^−/−^ but not in WT mice. Together, these data suggest that CCR7 plays a role in CD8^+^ ATT cell egress, but does not influence ATM accumulation or the metabolic impact of diet‐induced obesity.

## Introduction

Obesity has become a worldwide epidemic and increases risk for many diseases including diabetes, cardiovascular disease, and certain types of cancer. With regard to metabolic outcomes of obesity, the role of inflammation in various tissues has risen to the forefront of scientific investigation. Inflammatory immune cells, in particular macrophages, accumulate in adipose tissue (AT), muscle, liver, and brain in obese compared to lean animals (Weisberg et al. 2003; Xu et al. 2003; Fink et al. 2014; Kalin et al. 2015; Morinaga et al. 2015). AT macrophage (ATM) accumulation has also been demonstrated in obese humans (Bourlier et al. 2008; Wentworth et al. 2010). Various mechanisms have been proposed for the increased numbers of ATMs, including recruitment of monocytes from the circulation (Kanda et al. 2006; Huber et al. 2008), local proliferation (Amano et al. 2014; Haase et al. 2014), delayed egress (Ramkhelawon et al. 2014), and impaired turnover (Hill et al. 2015). The vast majority of research has been on recruitment‐mediated mechanisms – primarily on chemokines.

Approximately 50 chemokines and 20 chemokine receptors have been identified. Chemokines are small, 8–10 kDa proteins that act as chemoattractants. Because of their potent chemoattractant potential, many different chemokines and chemokine receptors have been knocked out or inhibited to determine their role in recruitment of immune cells to AT. Results of these studies have varied. Knockout studies targeting the most likely candidate for monocyte recruitment, CCL2, have demonstrated decreased (Kanda et al. 2006), no change (Inouye et al. 2007; Kirk et al. 2008), and even increased (Cranford et al. 2015) macrophages in AT. Studies have also been performed to determine the roles of CCL3 (Surmi et al. 2010), CCR5 (Kitade et al. 2012; Kennedy et al. 2013), and other chemokines/receptors (Nara et al. 2007; Chavey et al. 2009; Duffaut et al. 2009; Neels et al. 2009) in AT immune cell accumulation. Despite this focus on macrophage recruitment, the possibility of immune cell egress due to chemokine/chemokine receptors has not been fully investigated.

In addition to chemotactic signaling to attract immune cells to tissues, there are egress signals which provide cues for immune cells to exit the tissue and to enter neighboring draining lymph nodes where antigen presentation and T‐cell proliferation take place. The major function of this egress system is to regulate immunity and tolerance (Forster et al. 2008). CCL19, CCL21, and their receptor, CCR7, have been implicated in the egress of macrophages and T cells from multiple tissues [reviewed in (Hauser and Legler 2016)]. In addition, it has been shown that expression of these chemokine/receptor pairs is increased in AT of obese mice and a CCR7‐positive population of macrophages and T cells accumulates in obese AT (Lee et al. 2009; Zeyda et al. 2010). In a similar cellular retention system, netrin and Unc5b, normally studied for their neuroimmune guidance signaling, have recently been show to play a role in the retention of macrophages in AT (Ramkhelawon et al. 2014). We sought to determine whether CCR7 deficiency would alter ATM and T‐cell accrual and activation in AT.

## Methods and procedures

### Mice and diets

C57BL/6 wild‐type (WT) and CCR7^−/−^ mice on the C57BL/6 background were originally purchased from Jackson Laboratories (Bar Harbor, Maine). The CCR7^−/−^ mice were mated with WT mice and the heterozygous CCR7^+/−^ offspring were then crossed to generate the first wave of F2 littermate CCR7^−/−^ and WT mice used in our studies. Subsequent waves of mice were developed from CCR7^−/−^ mice that were intercrossed from F2 mice not used in studies or directly purchased from Jackson Laboratories. At 8 weeks of age, male mice were placed on 10% low‐fat diet (LFD; Research Diets #D12450D) or 60% high‐fat diet (HFD; Research Diets #D12492) for 16 weeks. Body weight was recorded weekly. Intraperitoneal glucose tolerance tests (GTT) were performed after 15 weeks of diet feeding, and all other analyses were performed after the mice were euthanized. All animal studies were performed after obtaining approval from the Vanderbilt Institutional Animal Care and Use Committee.

### Body composition

Lean body mass and body fat were quantified at the Vanderbilt University Mouse Metabolic Phenotyping Center via nuclear magnetic resonance (Bruker Minispec, Woodlands, TX).

### Fasting glucose, insulin, and glucose tolerance tests

Glucose tolerance was assessed in mice following a 5 h fast. Briefly, baseline fasting blood samples were obtained by cutting off the tip of the tail. Subsequently, blood samples were collected by massaging the tail 15, 30, 45, 60, 90, and 120 min after receiving an intraperitoneal injection of glucose (1 g/kg lean body mass). Blood glucose was measured using a Lifescan OneTouch Ultra glucometer (Johnson & Johnson, Northridge, CA). Fasting plasma insulin concentrations were measured using a commercially available ELISA according to manufacturer instructions (EMD Millipore, Billerica, MA).

### SVF isolation and flow cytometric analysis

The stromal vascular fraction (SVF) was isolated from epididymal fat pads via collagenase digestion and differential centrifugation as previously described (Orr et al. 2013). The following primary fluorophore‐conjugated antibodies, along with isotype controls, were used to characterize ATM and AT T‐cell (ATT) populations: APC‐conjugated anti‐mouse F4/80, FITC‐conjugated anti‐mouse CD11b, PE‐conjugated anti‐mouse CD11c, APC‐conjugated anti‐mouse TCR*β*, Alexa 700‐conjugated anti‐mouse CD4, PE‐conjugated anti‐mouse CD8a (all from eBioscience, San Diego, CA), and CF594 (Biotium, Fremont, CA)‐conjugated anti‐mouse CD163 (clone E10B10, provided by Cytoguide Aps, Aarhus, DK). Immediately prior to analysis, DAPI or propidium iodide was added to permit live/dead cell discrimination. Separate aliquots of SVF cells were used to characterize ATM and ATT cell populations. Flow cytometry was performed on a LSRFortessa flow cytometer (BD Biosciences, San Jose, CA) at the Vanderbilt Flow Cytometry Core Shared Resource, and data were analyzed using Cytobank.

### mRNA expression

RNA was isolated from epididymal AT using the RNeasy Mini Kit from Qiagen according to the manufacturer's instructions. cDNA was synthesized using the iScript cDNA synthesis kit also from Bio‐Rad, and real‐time RT‐PCR was performed on an iQ5 cycler (BioRad, Hercules, CA) using Taqman gene expression assays (Life Technologies, Carlsbad, CA; catalogue numbers available upon request). Expression of indicated genes was normalized to glyceraldehyde‐3‐phosphate dehydrogenase using the 2^−ΔΔCt^ method (Livak and Schmittgen 2001).

### Hepatic lipid accumulation

Livers were placed in OCT, frozen on dry ice, and stored at −20^o^ C until sectioned. Subsequently, Oil Red O staining of 10 *μ*m sections was performed to visualize hepatic neutral lipid accumulation, as previously described (Saraswathi et al. 2009). Liver triglyceride concentrations were measured using a commercially available colorimetric assay (Wako Diagnostics).

### Statistics

Statistical analyses were performed using GraphPad Prism software (version 6.05; GraphPad Software, La Jolla, CA). Repeated measures analysis of variance (ANOVA) with the Bonferroni multiple comparisons test was used to assess changes in dependent variables over time. The main effects of diet, genotype, and diet–genotype interactions were determined via two‐way ANOVA with the Tukey post hoc test. All data are reported and presented in figures as the mean ± SEM, and significance was set at *P *<* *0.05.

## Results

### CCR7 deficiency does not influence weight gain, hepatic lipid accumulation, or glucose tolerance during high‐fat feeding

**Figure 1:**
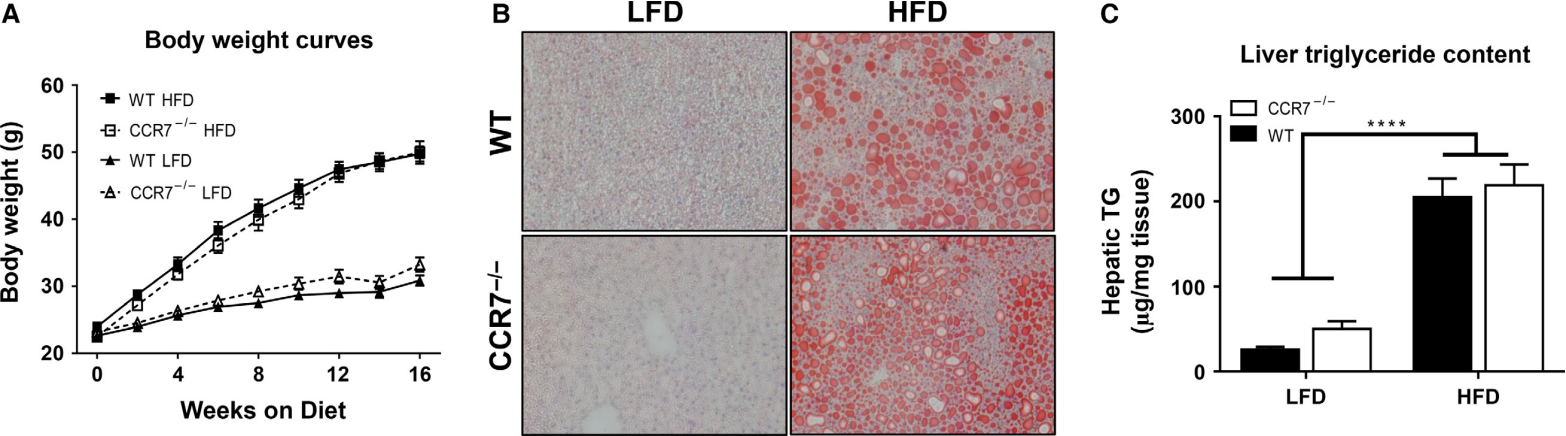
CCR7 deficiency does not influence weight gain or hepatic lipid accumulation during high‐fat feeding. (A) Growth curves of WT and CCR7^−/−^ mice fed low‐fat diet (LFD) or high‐fat diet (HFD) for 16 weeks starting at 8 weeks of age (*n *= 17–21/group). (B) Representative images of liver sections stained with Oil Red O to visualize neutral lipid accumulation. (C) Comparison of liver TG content from LFD and HFD fed WT and CCR7^−/−^ mice (*n *= 13–21/group, ****P <* *0.0001 for diet effect).

Male C57BL/6 mice were started on 10% LFD or 60% HFD at 8 weeks of age. The amount and rate of weight gain was similar between WT and CCR7^−/−^ mice over 16 weeks of LFD or HFD feeding (Fig. 1A). Likewise, hepatic neutral lipid and triglyceride accumulations following HFD feeding were not influenced by CCR7 deficiency (Fig. 1B and C). Body composition and organ weights were largely unaffected by genotype, although the proportion of lean body mass and spleen weight was significantly greater in CCR7^−/−^ mice compared to WT littermates regardless of diet (Table 1). In the C57BL/6 mice, HFD feeding increased AT expression of *Ccr7* by fivefold (*P* < 0.01), while *Ccl19* and *Ccl21a* were not altered (Fig. S1).

**Table 1:** Tissue weights. Body fat and lean body mass were quantified via nuclear magnetic resonance. Following sacrifice and central perfusion, epididymal adipose tissue, liver, and spleen were immediately harvested and weighed

Variable	LFD		HFD		Main effects
	WT	CCR7^−^/^−^	WT	CCR7^−^/^−^	
Body fat (g)	6.45 ± 0.57	6.30 ± 0.75	17.47 ± 0.91	18.43 ± 1.00	*P *<* *0.0001 for Diet
Lean body mass (g)	20.47 ± 0.29	22.49 ± 0.39	26.98 ± 0.55	27.29 ± 0.58	*P *<* *0.0001 for Diet *P *<* *0.05 for Genotype
Epididymal adipose tissue (g)	1.08 ± 0.10	1.04 ± 0.11	1.57 ± 0.09	1.89 ± 0.07	*P *<* *0.0001 for Diet
Liver (g)	1.23 ± 0.09	1.71 ± 0.11	2.51 ± 0.25	2.60 ± 0.19	*P *<* *0.0001 for Diet
Spleen (g)	0.09 ± 0.00	0.11 ± 0.01	0.12 ± 0.01	0.15 ± 0.01	*P *<* *0.001 for Diet *P *<* *0.01 for Genotype

**Figure 2:**
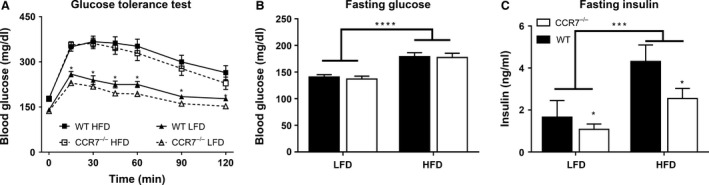
Glucose tolerance and fasting glucose and insulin concentrations. (A) Glucose excursion curves of low‐fat diet (LFD) and high‐fat diet (HFD) fed WT and CCR7^−/−^ mice during glucose tolerance test (*n *= 17–18/group, *P <* *0.05 for genotype effect for CCR7^−/−^ LFD versus WT LFD). (B) Comparison of fasting blood glucose concentrations of LFD and HFD fed WT and CCR7^−/−^ mice (*n *= 17–18/group, ****P <* *0.0001 for diet effect). (C) Comparison of fasting plasma insulin concentrations of LFD and HFD fed WT and CCR7^−/−^ mice (*n *= 11–17/group, ***P <* *0.001 for diet effect, *P <* *0.05 for genotype effect).

CCR7 deficiency did not impact glucose tolerance following HFD feeding; however, a significant main effect of genotype was observed in lean mice, with CCR7^−/−^ mice displaying reduced blood glucose concentrations throughout the GTT (*P *<* *0.05 for genotype effect; Fig. 2A). Although similar increases in fasting blood glucose concentrations were experienced by CCR7^−/−^ and WT mice on HFD (Fig. 2B), insulin concentrations were significantly reduced in both lean and obese CCR7^−/−^ mice compared to WT (Fig. 2C).

### CCR7 deficiency does not change ATM numbers

**Figure 3:**
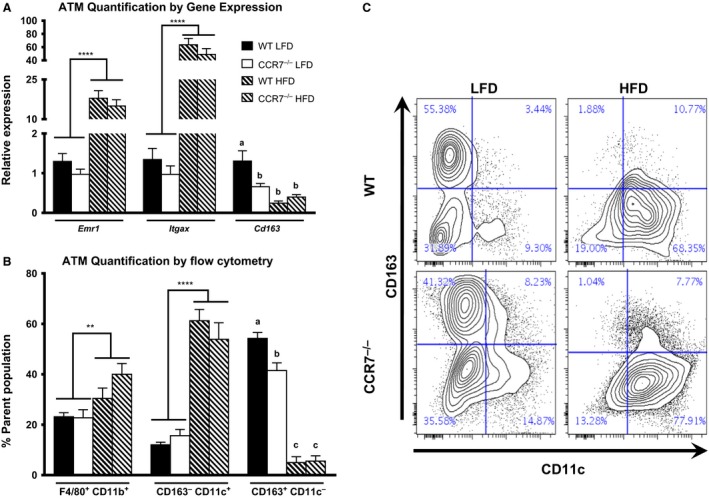
Adipose tissue (AT) macrophage quantification and polarization. (A) Comparison of AT mRNA expression of ATM markers in low fat diet (LFD) and high fat diet fed WT and CCR7^−/−^ mice (*n *= 9–14/group, ****P <* *0.0001 for diet effect, *P *<* *0.05 for groups not connected by the same letter). Please note, the WT LFD group was set as the reference group for each gene individually. (B) Quantification of ATM populations via flow cytometry. CD163^−^ CD11c^+^ and CD163^+^ CD11c^+^ populations are derived from the F4/80^+^ CD11b^+^ parent population (*n *= 6–8/group, ****P <* *0.0001 for diet effect, **P <* *0.01 for diet effect, *P *<* *0.05 for groups not connected by the same letter). (C) Representative flow plot of CD163 and CD11c expression from F4/80^+^ CD11b^+^ ATMs.

HFD feeding was accompanied by a significant increase in AT mRNA expression of *Emr1* (F4/80) and *Itgax* (CD11c; Fig. 3A), providing indirect evidence of M1 ATM accumulation in both CCR7^−/−^ and WT mice. A significant interaction effect was detected for AT expression of the M2 marker *Cd163*, with LFD‐fed WT mice displaying significantly greater expression compared to all other groups (Fig. 3A). Consistent with AT mRNA expression, direct quantification of ATM populations via flow cytometry confirmed the accumulation of ATMs (F4/80^+^CD11b^+^; Fig. 3B and C) in HFD‐fed mice, which were overwhelmingly M1 polarized (i.e., CD163^−^CD11c^+^; Fig. 3B and C). Additionally, the proportion of M2 ATMs (i.e., CD163^+^CD11c^−^) was greatest in LFD‐fed WT mice; however, LFD‐fed CCR7^−/−^ mice also displayed significantly greater proportion of CD163^+^CD11c^−^ ATMs compared with HFD‐fed WT and CCR7^−/−^ (Fig. 3B and C). *Plin2* and *Abca1* are two genes associated with a metabolically activated phenotype of macrophages in obesity (Kratz et al. 2014). While *Abca1* was not altered among the groups in our studies, *Plin2* was fivefold elevated in the HFD‐fed mice (*P* < 0.001), without an effect of genotype (Fig. S2).

### CCR7 deficiency induces CD8^+^ ATT cell accumulation and augments AT inflammation during HFD feeding

**Figure 4:**
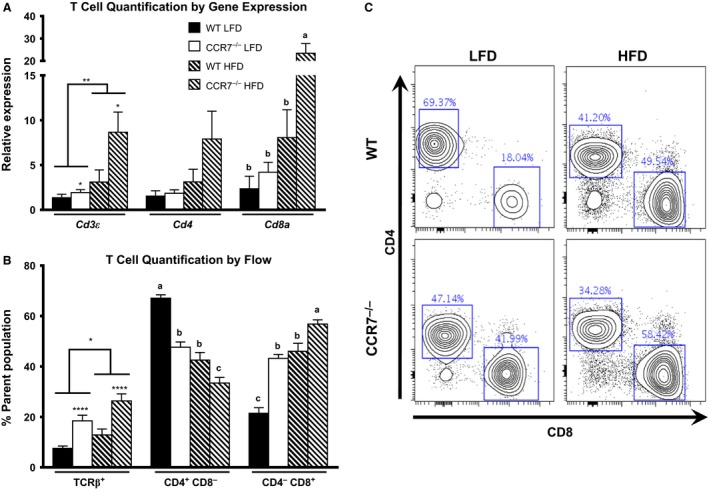
Adipose tissue (AT) T‐cell quantification. (A) AT mRNA expression of ATT markers (*n *= 9–14/group, **P <* *0.01 for diet effect, *P <* *0.05 for genotype effect, *P *<* *0.05 for groups not connected by the same letter). (B) Quantification of flow cytometry data (*n *= 8–14/group, ****P <* *0.0001 for genotype effect, *P <* *0.05 for diet effect, *P *<* *0.05 for groups not connected by the same letter). (C) Representative flow plot of CD4 and CD8 from TCR *β*+ ATTs.

**Figure 5:**
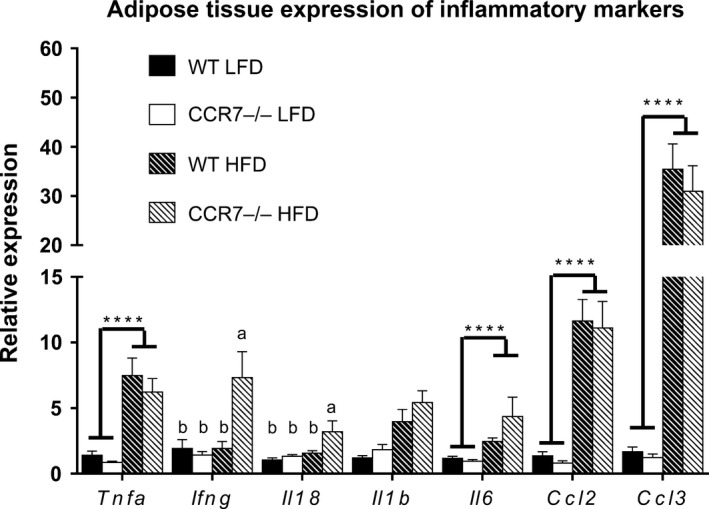
Adipose tissue (AT) inflammation. Comparison of AT mRNA expression of inflammatory chemokines and cytokines in low‐fat diet and high‐fat diet fed WT and CCR7^−/−^ mice (*n *= 9–14/group (except for Il18 where *n *= 5/gp), ****P <* *0.0001 for diet effect, **P <* *0.01 for diet effect, *P *<* *0.05 for groups not connected by the same letter).

With regard to ATTs, significant diet and genotype effects were detected for AT expression of *Cd3ε*; HFD feeding and CCR7 deficiency increased *Cd3ε* expression (Fig. 4A). Likewise, HFD feeding and CCR7 deficiency induced a significant accumulation of ATT cells based on flow cytometric analyses (Fig. 4A). In contrast to the marginal impact of CCR7 deficiency on the distribution of ATM subpopulations, CCR7 deficiency induced a preferential accumulation of CD8^+^ ATT cells. A significant increase in AT *Cd8a* expression was detected in HFD‐fed CCR7^−/−^ compared to HFD‐fed WT mice. A flow cytometric analysis of the AT SVF indicates that CD8^+^ ATT cell accumulation was significantly greater in CCR7^−/−^ mice compared to WT counterparts on both LFD and HFD (Fig. 4B and C). Additionally, the proportion of CD4^+^ ATT cells was significantly reduced in CCR7‐deficient mice compared to diet‐matched WT counterparts (Fig. 4B and C). The difference in flow cytometry versus gene expression for CD4 likely reflect an overall increase in CD4 ATTs but a relative reduction as a percent of the TCR*β* ^+^ cells due to the even larger increase in CD8 ATTs. With regard to AT inflammation, a significant diet effect was observed for AT mRNA expression of *Tnf, Il6, Il1b, Ccl2, and Ccl3* (Fig. 5). A significant diet–genotype interaction was detected for *Ifng and Il18*, with HFD‐fed CCR7^−/−^ mice displaying significantly greater expression compared to LFD‐fed mice and HFD‐fed WT (Fig. 5).

## Discussion

The progressive accumulation of inflammatory ATMs is a hallmark of diet‐induced obesity (DIO) (Hill et al. 2014). Although a great deal of research has focused on the signals responsible for monocyte recruitment and subsequent ATM differentiation and polarization in obesity, the potential contribution of altered retention and egress signals has received little attention. The goal of this study was to determine the impact of reduced AT leukocyte emigration on ATM accumulation, glucose metabolism, and AT inflammation in DIO. Because of previous studies demonstrating the role of CCR7 in leukocyte egress from sites of inflammation to secondary lymphoid organs, we utilized CCR7 knockout mice for these studies (Forster et al. 2008). We hypothesized that deleting CCR7 would reduce the capacity for ATMs to egress and subsequently decrease antigen presentation, thus disrupting T‐cell activation and recruitment to the AT. However, an alternative hypothesis was that CCR7 deficiency would directly reduce the egress of T cells, resulting in their accumulation. Our results suggest that the latter is true (i.e., we detected an increase in ATTs without any change in ATMs).

The primary finding of this study is that CCR7 deficiency significantly alters leukocyte populations within the AT. Most notably, CCR7‐deficient mice display a significant increase in the accumulation of CD8^+^ ATT cells. Additionally, the proportion of M2 ATMs is significantly reduced in LFD‐fed CCR7^−/−^ mice. Despite the observed differences in AT leukocyte populations, CCR7 deficiency did not have any discernable impact on the metabolic consequences of DIO. Specifically, HFD‐fed CCR7^−/−^ and WT mice did not differ with respect to weight gain, body fat, hepatic steatosis, or glucose tolerance. Likewise, AT inflammation was largely unaffected by CCR7 deficiency; only *Ifng* and *Il18* expression were significantly increased in HFD‐fed CCR7^−/−^ mice compared to WT counterparts. It is interesting to note that although glucose tolerance was not influenced by genotype, fasting glucose, and insulin concentrations were significantly reduced in CCR7^−/−^ mice, suggesting that CCR7 deficiency may improve basal insulin action. These data are difficult to explain, as the only immunophenotype we noted was an increase in AT CD8^+^ T cells – a condition that would be expected to decrease insulin action (Kintscher et al. 2008; Nishimura et al. 2009). It is possible that this slight improvement in metabolic phenotype is due to changes in perinodal AT inflammation and reduced homing of dendritic cells to lymph nodes as was recently reported by Hellmann et al. (2016) and discussed in more detail below. Another possibility is that the CCR7^−/−^ mice have reduced insulin secretion due to changes in their pancreas. For example, in a model of type 1 diabetes, desensitization to CCR7 blocked T‐cell migration into islets (Shan et al. 2014). To our knowledge, a role for CCR7 and T cells in the pancreas in type 2 diabetes has not been studied.

Our studies demonstrated that CCR7 deficiency did not alter ATM accumulation in HFD‐fed mice. Although CCR7 has been shown to play an important role in the migration of CD11b^+^CD11c^+^ dendritic cells (DC) from peripheral tissues to draining lymph nodes, CCR7 deficiency does not completely abolish DC migration. It is possible that, in the absence of CCR7, other chemokines, such as CXCL12, may play a compensatory role (Kabashima et al. 2007; Ricart et al. 2011). Additionally, Ramkhelawon et al. (2014) recently demonstrated that HFD feeding impairs CCR7‐mediated ATM chemotaxis due to elevated netrin‐1 expression. Preventing netrin‐1 signaling in ATMs via adoptive transfer of *Ntn1* ^−*/*−^ fetal liver cells, increases ATM emigration to draining lymph nodes, suggesting that CCR7 remains a viable target for altering ATM accumulation in obesity.

The most prominent impact of CCR7 deficiency was the increased accumulation of CD8^+^ ATT cells during HFD feeding, which has previously been implicated in obesity associated AT inflammation and insulin resistance subsequent to ATM recruitment (Nishimura et al. 2009). Our data suggest that CD8^+^ ATT cell accumulation is not a primary causative event and are consistent with a recent study by Cho et al. (2014), in which whole body and macrophage specific MHCII deficiency attenuated insulin resistance and AT inflammation in HFD‐fed mice despite showing a significantly greater accumulation of CD8^+^ ATT cells. The preferential accumulation of CD8^+^ ATT cells, as opposed to CD4^+^ ATT cells in CCR7^−/−^ mice is somewhat surprising, as CCR7 has been shown to play a role in tissue egress of both CD8^+^ and CD4^+^ T cells (Jennrich et al. 2012; Gómez et al. 2015). However, there is also evidence that CD4^+^ T cells do not require CCR7 for egress (Vander Lugt et al. 2013). Overall, our data support the latter, i.e., that CCR7 is involved in CD8 T cell – but not CD4 T cell – egress from AT.

This is not the first study to investigate the impact of CCR7 deficiency on DIO. Recently, Sano et al. (2015) reported that CCR7^−/−^ mice were protected from weight gain and insulin resistance following HFD feeding, with a dramatic decrease in the mRNA expression of macrophage markers in their AT. One important difference may be the relatively small sample size in the study by Sano et al. (i.e., 3–5 per group). Furthermore, it is unclear whether mice used in their study were littermates or derived from separate colonies. Genetic drift or slight differences in the background strain can significantly impact weight gain and AT inflammation (Attie and Keller 2010). We have highlighted these important study design elements, as well as the importance of cohousing mice from different groups to remove the effects of differing microbiota and matching mice for initial body weight, in a recent commentary (Hasty and Gutierrez 2014). Nonetheless, the most likely reason for differences in AT inflammation, hepatic steatosis, and systemic insulin resistance between the two studies (i.e., large reduction in the study by Sano et al. and almost no change in our study) is the lack of weight gain in the CCR7^−/−^ mice in their experiments, as weight gain is a primary driver of these pathologies. Interestingly, despite these differences, Sano et al. also detected an increase in expression of T‐cell‐related genes, such as CD3, CD4 and CD8, in the AT of their CCR7^−/−^ mice. While our manuscript was in revision, Hellmann et al. (2016) also published their work on CCR7 and AT inflammation. In very thorough studies, they showed that *Ccr7* deficiency prevented the accumulation of CD11c^+^ cells in regional lymph nodes and also that CCR7^−/−^ mice had reduced numbers of T cells, B cells, and macrophages in their AT. Similar to our studies and opposed to Sano et al., their studies showed no impact of CCR7 deficiency on weight gain or fat mass; however, the CCR7^−/−^ mice had a slight improvement in glucose tolerance and a reduction in fasting plasma insulin – the insulin data being similar to what we report. A nice element included by Hellmann et al. was the analysis of CD11c^+^ cells in lymph nodes and perinodal AT of their mice, where they found that obesity increases the antigen presenting cells.

Overall, results of the present study suggest that CCR7 plays an important role in CD8^+^ ATT cell trafficking; however, the physiological relevance of this observation remains unclear, as the preferential accumulation of CD8^+^ ATT cells in CCR7‐deficient mice had no discernable metabolic impact. Although our data do not support an obvious role for CCR7 in regulating ATM egress, future studies should explore the extent to which retention signals, such as netrin‐1, function by inhibiting CCR7‐mediated migration.

## Supporting information




**Figure S1.** Gene expression of CCR7, CCL19, and CCL21 in adipose tissue of LFD versus HFD fed mice.
**Figure S2.** Gene expression of Abca1 and Plin2 in adipose tissue of LFD versus HFD fed WT and CCR7−/− mice. ****P < 0.001 for diet effect.
