# Supplementary material for: CC‐chemokine receptor 7 (CCR7) deficiency alters adipose tissue leukocyte populations in mice
Source: Physiol Rep. 2016 Sep 21;4(18):e12971. doi: 10.14814/phy2.12971 (PMC5037919; doi:10.14814/phy2.12971)
Supplement: Supplementary file 1 — Figure S1. Gene expression of CCR7, CCL19, and CCL21 in adipose tissue of LFD versus HFD fed mice. Figure S2. Gene expression of Abca1 and Plin2 in adipose tissue of LFD versus HFD fed WT and CCR7−/− mice. ****P < 0.001 for diet effect. [file PHY2-4-e12971-s001.pptx]

## Slide 1
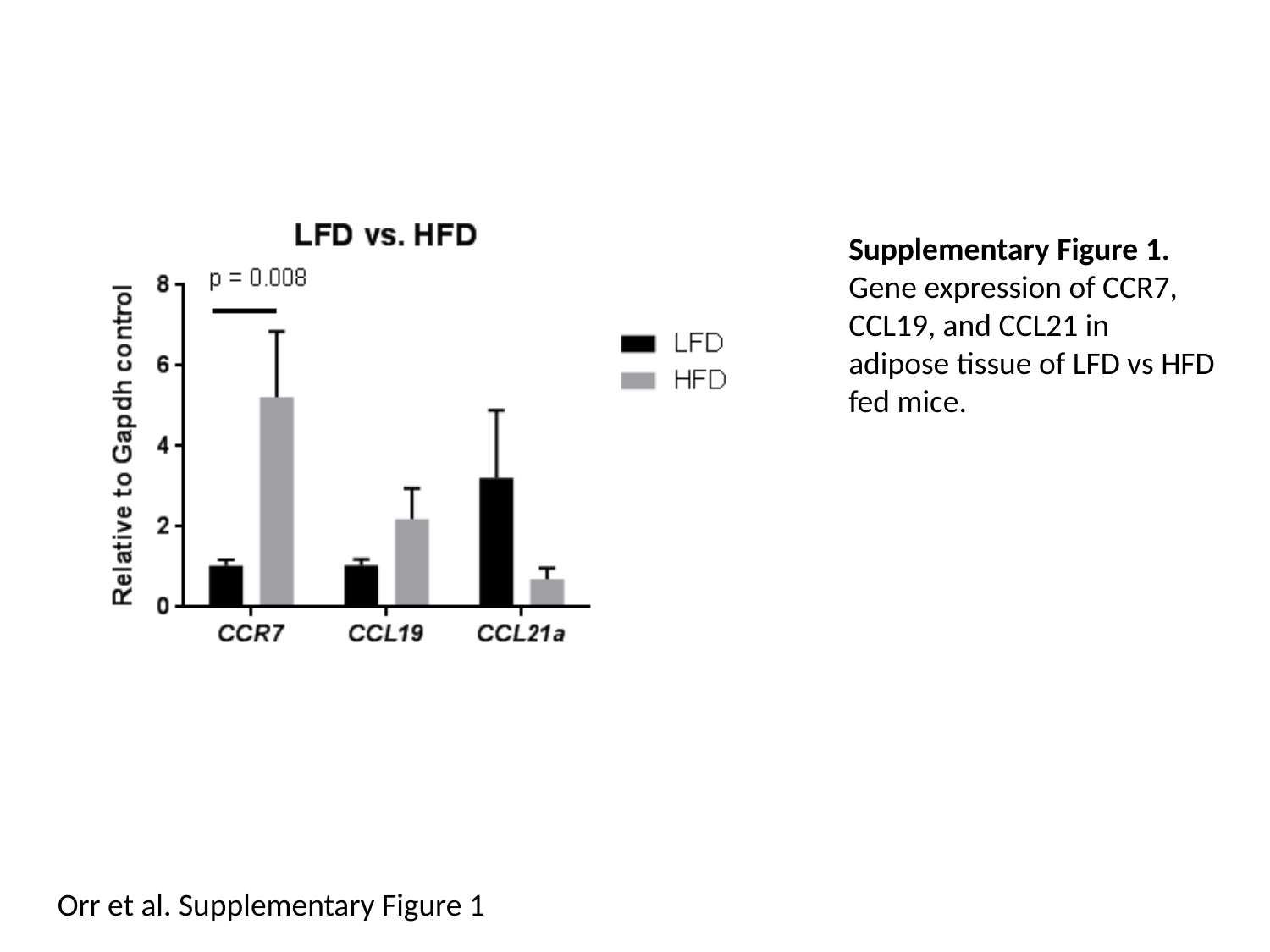

Supplementary Figure 1.
Gene expression of CCR7, CCL19, and CCL21 in adipose tissue of LFD vs HFD fed mice.
Orr et al. Supplementary Figure 1

## Slide 2
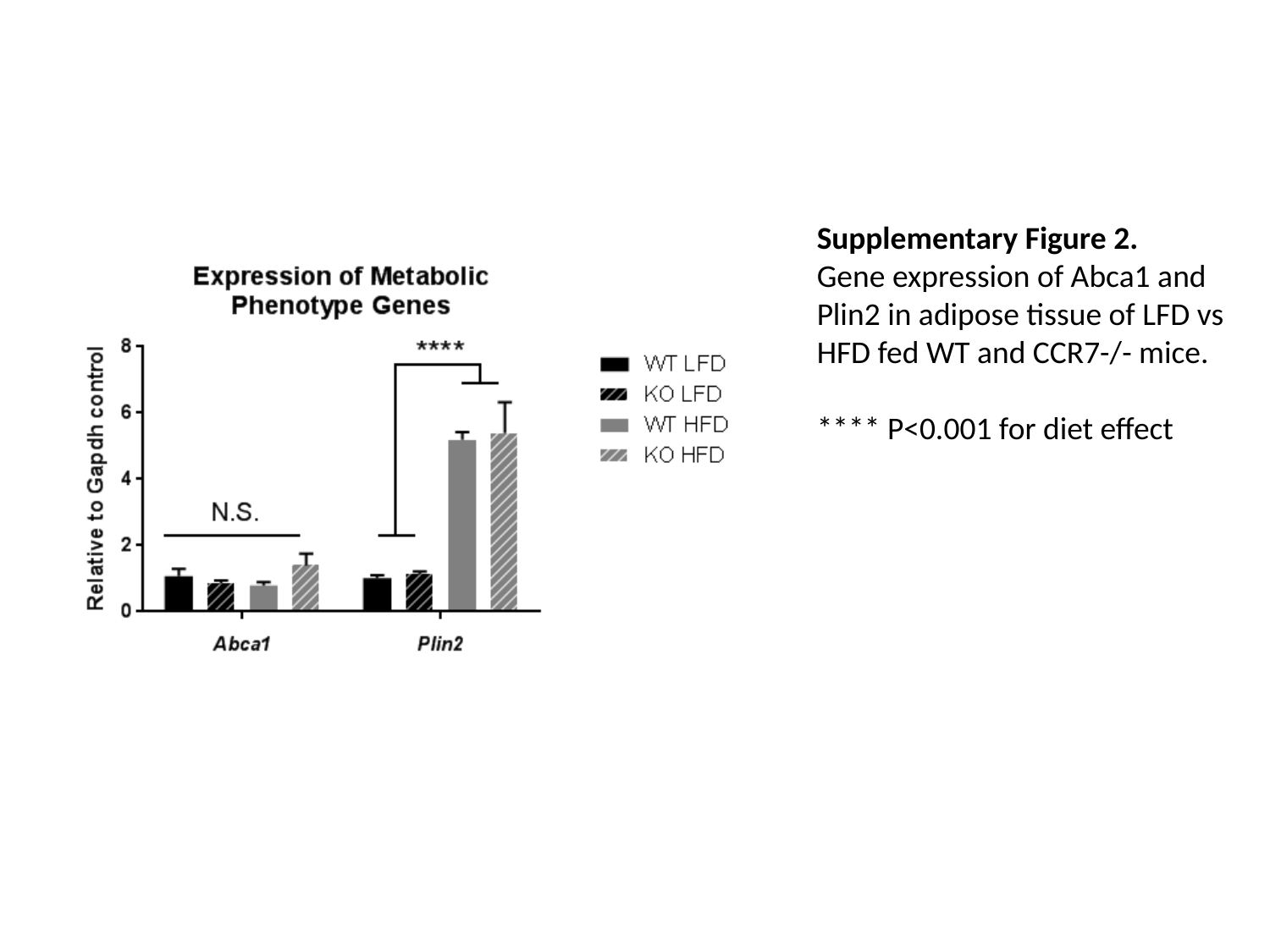

Supplementary Figure 2.
Gene expression of Abca1 and Plin2 in adipose tissue of LFD vs HFD fed WT and CCR7-/- mice.
**** P<0.001 for diet effect
